# Evolution of Cochlear implant mapping and vestibular function in a pediatric case of Labyrinthitis

**DOI:** 10.1186/s40463-020-0403-2

**Published:** 2020-02-05

**Authors:** Sophie Lipson, Ross O’Shea, Susan Gibbons, Guangwei Zhou, Jacob Brodsky

**Affiliations:** 1 Department of Otolaryngology and Communication Enhancement, Boston Children’s Hospital, 300 Longwood Avenue, Boston, MA 02115 USA; 2 Department of Otolaryngology, Harvard Medical School, 25 Shattuck Street, Boston, MA 02115 USA

**Keywords:** Cochlear implant, Labyrinthitis, Vestibular disorder, Pediatric

## Abstract

**Background:**

Vestibular symptoms such as vertigo and imbalance are known to occur in some cochlear implant patients during the immediate postoperative period; however, acute vertigo in implanted children occurring remotely from the postoperative period has not been previously well-described.

**Case presentation:**

A three-year-old girl with a history of bilateral sequential cochlear implantation presented with acute labyrinthitis associated with sudden onset of vertigo, balance impairment, and decline in right cochlear implant function 2 years after her most recent implant surgery. We describe her audiological and vestibular testing results during both the acute phase and following medical management and recovery.

**Conclusion:**

Acute labyrinthitis should be considered when sudden onset vertigo and/or imbalance presents in children with cochlear implants outside of the perioperative period. Such symptoms should prompt early assessment of cochlear implant function, so that the device can be reprogrammed accordingly.

## Background

Vestibular loss is common in children with sensorineural hearing loss and with cochlear implants (CI) [1]. Further impairment of vestibular function and associated postoperative vestibular symptoms are also known risks of cochlear implant surgery [2–4]. However, the onset of vestibular symptoms outside of the immediate perioperative window has not been well-described. Included in the differential diagnosis of such symptoms is the diagnosis of acute labyrinthitis, which may include a deterioration in residual acoustic hearing or decline in implant function. Early identification of labyrinthitis in a child with a cochlear implant is important, because children may not report an associated decline in CI function. We describe a case of a child with acute labyrinthitis remote from the perioperative period, which was detected early enough to allow us to obtain vestibular testing and implant function testing during both the acute phase and following recovery, which yields an ideal opportunity to shed light on this important entity.

## Case presentation

A three-year-old girl with bilateral profound congenital sensorineural hearing loss of unknown etiology underwent right cochlear implantation (CI) at 12 months old and left cochlear implantation at 16 months old. Both implant surgeries were uneventful. Preoperative magnetic resonance imaging showed unremarkable cochlear and vestibular anatomy. Preoperative cervical vestibular evoked myogenic potential (VEMP) testing was normal.

**Fig. 1:**
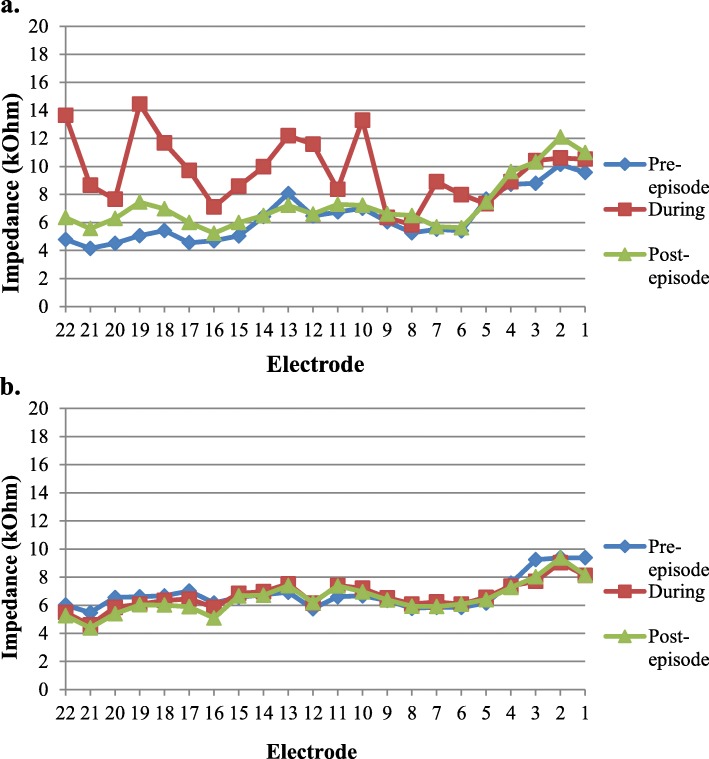
Electrode impedance levels measured before, during, and after onset of labyrinthitis. **a**. Right ear (affected), **b**. Left ear (unaffected)

**Fig. 2:**
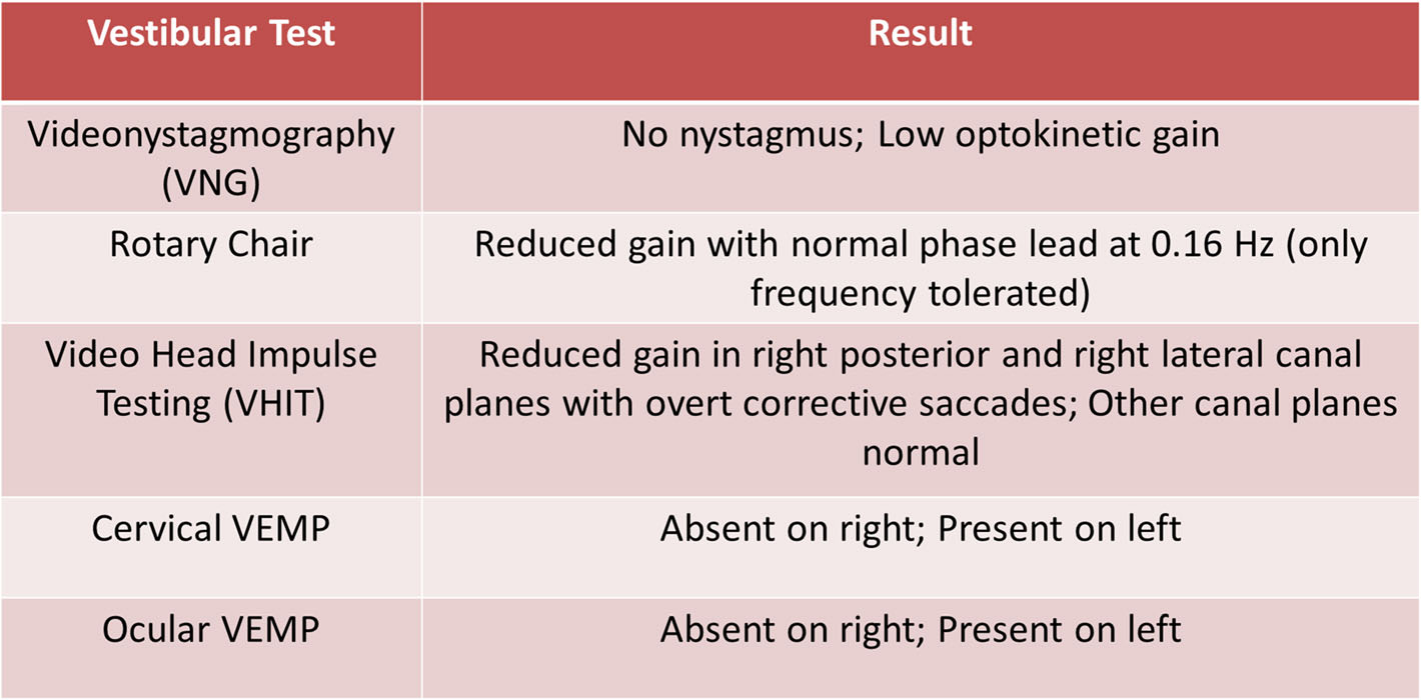
Vestibular testing results 8 days after onset of episode of labyrinthitis

Two years post-implantation, the patient experienced the sudden onset of room-spinning vertigo and imbalance, accompanied by a parent-reported change in hearing performance. Cochlear implant programming 4 days after symptom onset demonstrated an increase in right-sided impedances, resulting in non-compliance across 4/22 channels (Fig. 1). Device responses indicated the need for reprogramming across all channels for the right side. Tympanometric results were within normal limits bilaterally, and her ear exam was normal bilaterally without evidence of effusions. A 3-week prednisone taper was initiated with a starting dosage of 1 mg/kg. The patient underwent vestibular testing 8 days after symptom onset, including rotary chair, cervical and ocular VEMP, and video head impulse testing (vHIT). Results showed evidence of severe right-sided peripheral vestibular loss (Fig. 2). The acute onset of combined unilateral cochlear and vestibular impairment involving all five vestibular organs was consistent with a diagnosis of acute labyrinthitis.

All symptoms resolved entirely by 10 days post-onset. At 29 days post-onset, cochlear implant programming demonstrated that right impedance and stimulation levels returned to baseline. Audiological testing indicated baseline right CI soundfield puretone thresholds and word recognition scores. There were no residual balance deficits on clinical examination 4 weeks after symptom onset. Vestibular retesting 1 year later showed a recovery of peripheral vestibular function, except for continued absence of ocular VEMP responses.

## Discussion

Acute labyrinthitis in the setting of a cochlear implant has been described in a limited number of cases in adults, but this is the first pediatric case reported to our knowledge. Itayem and colleagues were the first to describe the clinical and electrophysiological constellation in adults, which they refer to as “cochlear implant associated labyrinthitis.” [5] They reported acute onset dizziness, device performance decline, and a characteristic erratic pattern of electrode impedances occurring after an asymptomatic postoperative period in adult CI recipients. The exact etiology of this presentation remains unknown, however our case is suggestive of a non-suppurative inflammatory cause. This is supported by the complete resolution of symptoms along with return of impedance and stimulation levels to baseline following prompt initiation of corticosteroids and the absence of other signs of infection (e.g. fever, ear pain, etc.).

Endocochlear inflammation has been implicated in other cases of suspected soft device failure. Benatti et al. reported a case of gradual performance decline plus acute onset facial nerve paralysis in an eight-year-old CI recipient, ultimately necessitating explantation [2]. Histological analysis confirmed the presence of fibrotic tissue around the electrode array [2]. Treatment with corticosteroids provided some transitory improvement in symptoms in that case. The patient was also noted to have IgM specific for paramyxovirus type 1, which subsequently became negative 1 month later. This also raises the suspicion of a viral antecedent. Wolfe and colleagues furthered the inflammation theory in their case of apparent device failure in a 75-year-old CI recipient with increasing electrode impedance [6]. They proposed that this was due to changes in the dynamic electrochemical properties of cochlear fluids and the physical properties of surrounding tissues rather than faults in the physical characteristics of the electrode leads and contacts. Explantation was performed due to suspected device failure; however, upon activation of the replacement implant, erratic impedance patterns and performance decline were once again encountered. Interestingly, the patient was found to have a paradoxical decrease in impedance patterns after a period of prescribed non-use, followed by recurrence of erratic increased impedance patterns upon reactivation. The authors therefore suggested the concept of “electro-toxic inflammation” and recommended refraining from increasing current amplitude to provide sufficient loudness growth for a recipient who is experiencing difficulty secondary to a change in electrode impedance. This patient was commenced on systemic corticosteroid treatment, which was followed by immediate and dramatic improvement in impedances. Although the vestibular system did not appear to be affected in either of these examples, it is conceivable that extension of this inflammatory reaction may result in a confluent cochleitis/labyrinthitis, as illustrated by our case.

This case highlights the utility of preoperative vestibular testing in the pediatric CI population, in which vestibular and balance impairment is common [1, 3, 7]. The differential diagnosis of acute vestibular symptoms in pediatric patients is broad, which can make accurate diagnosis difficult [8]. Moreover, pediatric patients often lack the developed language skills to accurately express themselves, further underscoring the importance of objective vestibular testing within this cohort. Although sometimes challenging to obtain, vestibular testing is feasible in young children and could aid in the timely diagnosis and management of pediatric patients with vestibular symptoms and balance problems. Jacot et al. found in a cohort of 224 patients undergoing CI for sensorineural hearing loss that only 50% of patients had preoperatively normal bilateral vestibular function [3]. Therefore, preoperative baseline vestibular testing may be useful in identifying and managing later-onset vestibular and balance deficits for pediatric CI patients. Access to vestibular testing results in the current case at baseline and during the acute labyrinthitis and recovery phases was useful in supporting the diagnosis and tracking recovery, as well as in gaining insight into the pathophysiologic mechanisms at play. However, this diagnosis likely could have been made in the absence of vestibular testing, and the priority was the assessment and reprogramming of the device.

## Conclusions

Labyrinthitis should be considered when acute onset vertigo occurs in children with cochlear implants remote from the perioperative period. Early device reprogramming and administration of systemic corticosteroids are interventions that may help to expedite recovery. Further research is needed to expand our knowledge of this presentation with regard to etiology and optimal management.

## Data Availability

Data sharing is not applicable to this article as no datasets were generated or analyzed during the current study.

## Abbreviations

CI: Cochlear Implant (ation)
VEMP: Vestibular Evoked Myogenic Potential
VHIT: Video Head Impulse Test
